# Supplementary material for: Scaffold Pore Curvature Influences ΜSC Fate through Differential Cellular Organization and YAP/TAZ Activity
Source: Int J Mol Sci. 2022 Apr 19;23(9):4499. doi: 10.3390/ijms23094499 (PMC9102667; doi:10.3390/ijms23094499)
Supplement: Supplementary file 1 [file ijms-23-04499-s001.zip › ijms-1635854 (2).pdf]

Table S1. Primers for qPCR gene expression analysis.

GENE	FORWARD	REVERSE
**CD44**	GTGGCACACAGCTTGGGGA	TCAGAGCCAGTGCCAGGAGAGAT
**COL1A1**	GCTCCTCTTAGGGGCCACT	CCACGTCTCACCATTGGGG
**RUNX2**	TGGCCGGGAATGATGAGAAC	TGAAACTCTTGCCTCGTCCG
**SP7**	GGGCGTTCTACCTGCGACTG	ATCGGGGCGGCTGATTG
**GAPDH**	CGTCCCGTAGACAAAATGGT	TTGATGGCAACAATCTCCAC
**CTGF**	GGCCTCTTCTGCGATTTCG	GCAGCTTGACCCTTCTCGG
**YAP1**	ACCCTCGTTTTGCCATGAAC	TGTGCTGGGATTGATATTCCGTA
**AREG**	GGTCTTAGGCTCAGGCCATTA	CGCTTATGGTGGAAACCTCTC
**VCL**	TCTCGCACCTGGTGATTATGC	TGAACAGTCTCTTTTCCAACCC
**ZYX**	CCGATGATCGAGGAACCATTC	CGTTCTTGGTCATGTCGTCCA
**TLN1**	TACTACATGCTCCGAAATGGGG	CACCGTTCCGTCTAACATCCG
**TLN2**	GCCACTGCAATGTGGTGAAG	TCTCCCTAATGACTCGACAAGC
**RHOA**	GAAACTGGTGATTGTTGGTGATG	ACCGTGGGCACATAGACCT
**GPRCA5**	ACCACAGACTTTGTGACCTGG	CGAGTGCAAACATGCAAGCC
**MCAM**	CCCAAACTGGTGTGCGTCTT	GGAAAATCAGTATCTGCCTCTCC
**KDNL2**	TGGACGGGATAAGGATGCCA	TGACATCGAGTTTTTCCACCAAC
**LMNA**	GGATGCTGAGAACAGGCTACA	GCTTGGCGGAGTATGTCTTTT
**NCAD**	AGGCTTCTGGTGAAATTGCAT	GTCCACCTTGAAATCTGCTGG
**CX45**	AGATCCACAACCATTCGACATTT	TCCCAGGTACATCACAGAGGG
**NESTIN**	CCCTGAAGTCGAGGAGCTG	CTGCTGCACCTCTAAGCGA
**CX23**	GAAGGATGTGTTAAGCCTCCAA	CTCATTCCCGTAGACAGCAAAG

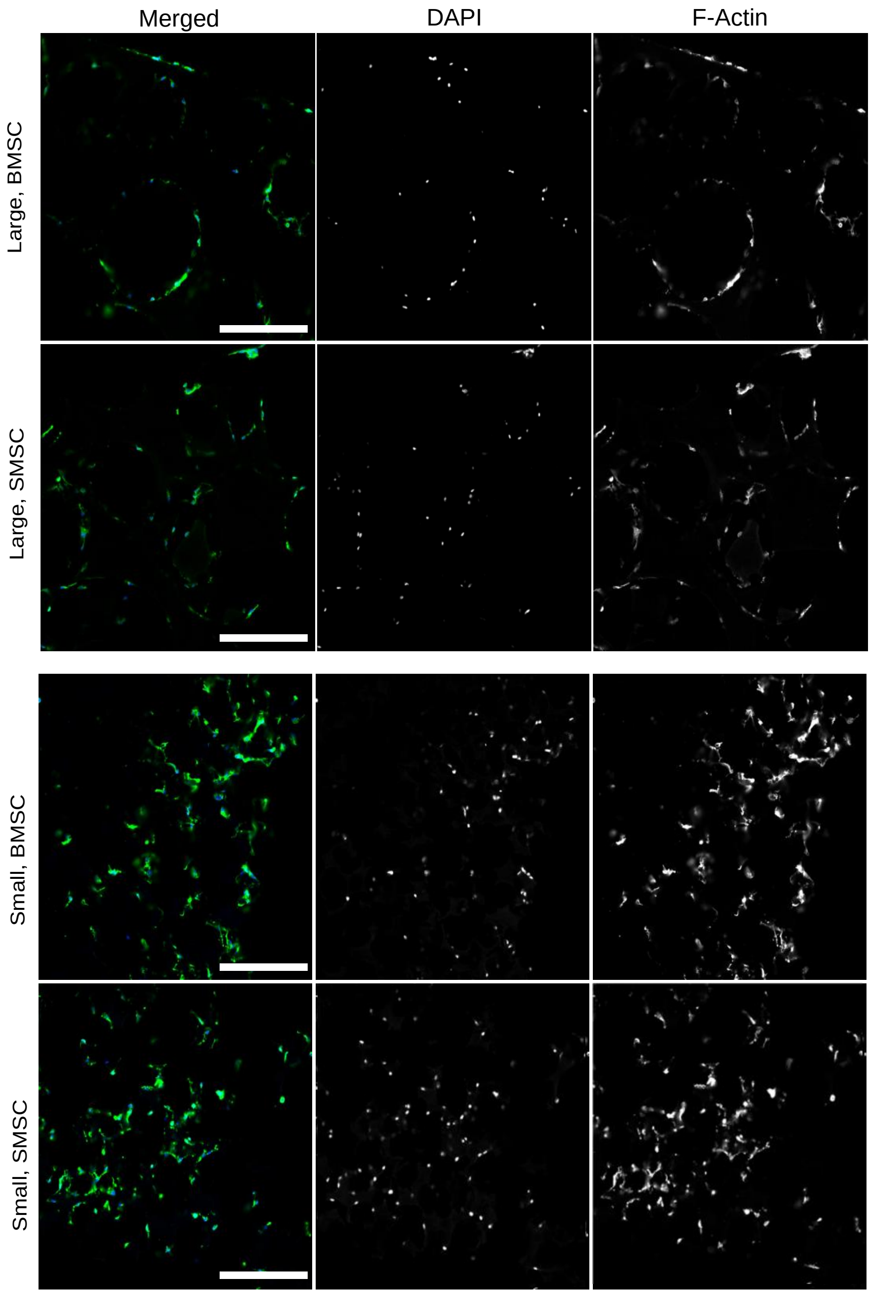

Figure S1. Confocal laser microscopy of cell-seeded scaffolds cultured for 1 week in vitro demonstrate uniform cell seeding density at low magnification (green: actin, blue: DAPI/nucleus; scale = 250 um).

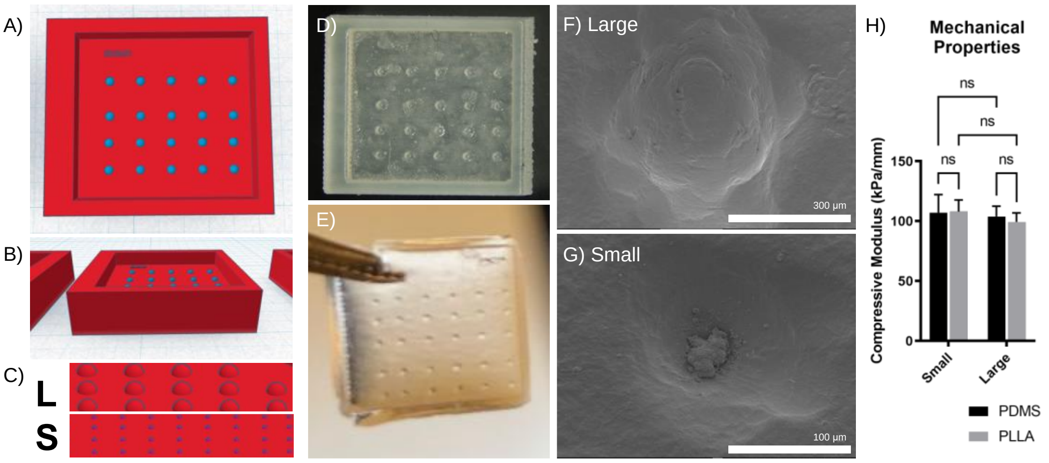

Figure S2. Pore hemisphere tools were designed in Tinker CAD (**A**–**C**) and 3D printed (**D**). Imaging tools were cast in polydimethylsiloxane (PDMS) by soft lithography and cured by heat-induced polymerization (**E**). The surface morphology and pore size of PDMS constructs (laden with cells) is visualized by scanning electron microscopy (**F**,**G**). PDMS synthesis was optimized to match the mechanical properties of PLLA scaffolds (H, all combinations are non-significant (n.s.)).

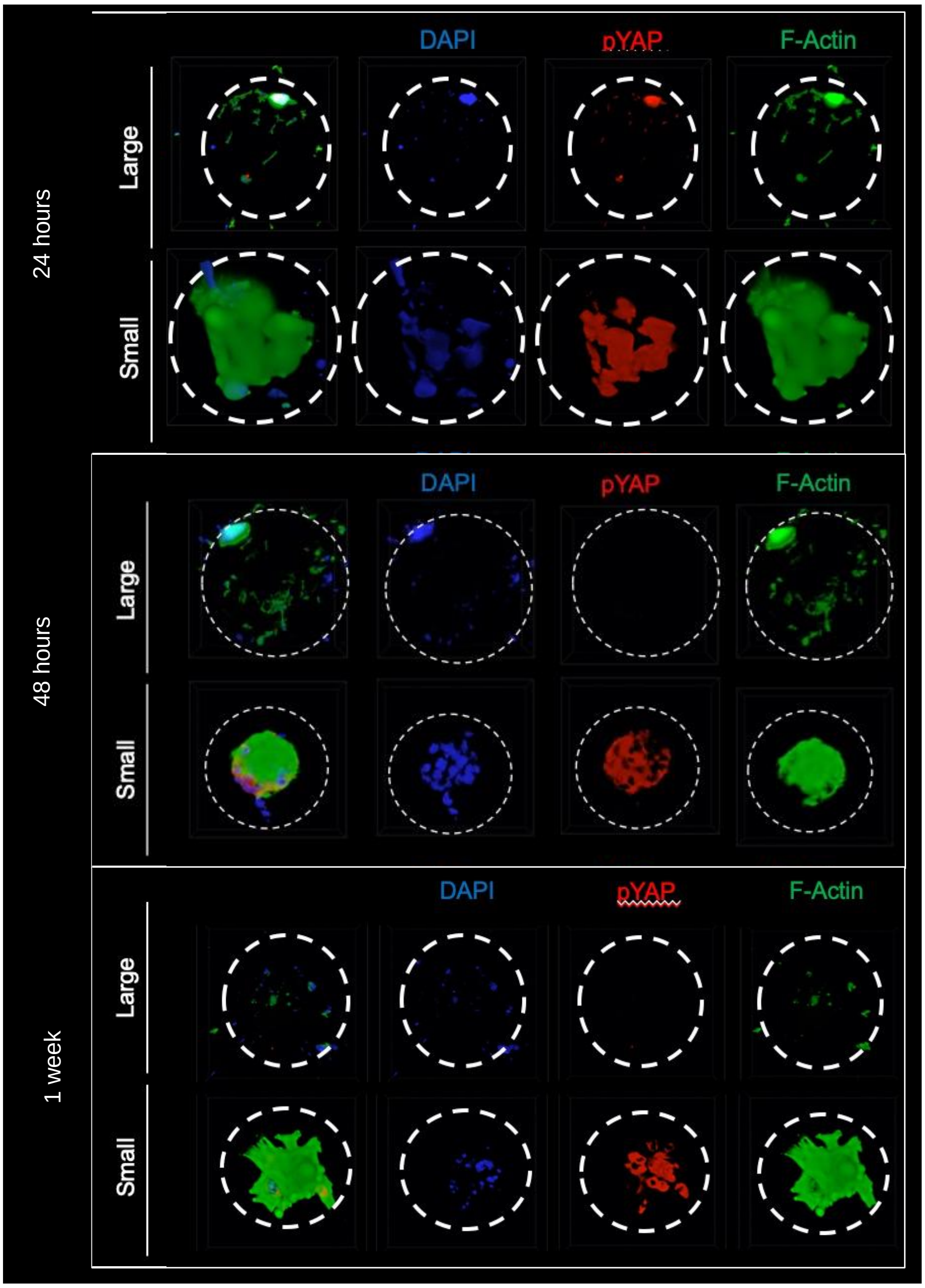

Figure S3. Suture mesenchymal stem cells (SMSCs) are cultured on PDMS macropore-mimetic hemispheres for up to 1 week in vitro; changes in YAP phosphorylation (red) are observed by confocal laser microscopy and immunofluorescence. Hemisphere perimeters are outlined in white.

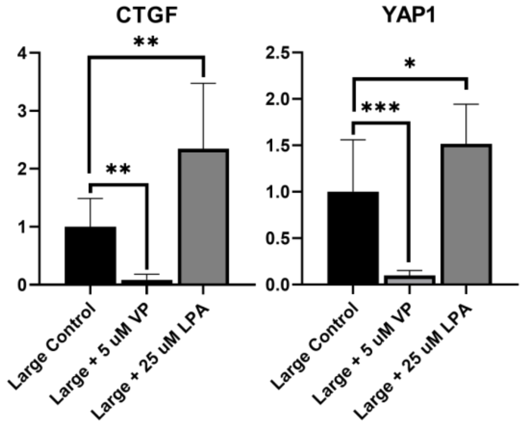

Figure S4. Pharmacologic administration of verteporfin (VP, 5 uM) and lysophosphatidic acid (LPA, 25 uM) inhibit and activate YAP signaling, respectively, compared to vehicle control. * = $p < 0.05$, ** = $p < 0.01$, *** = $p < 0.001$, **** = $p < 0.0001$.
